# Sunlight Exposure and Vitamin D Levels in Older People-An Intervention Study in Swedish Nursing Homes

**DOI:** 10.1007/s12603-020-1435-z

**Published:** 2020-07-10

**Authors:** Maria Samefors, A. Tengblad, C.J. Östgren

**Affiliations:** 1 Department of Health, Medicine and Caring Sciences, Linköping University, SE 581 83, Linköping, Sweden; 2 Wetterhälsan, Jönköping, Sweden

**Keywords:** Older people, nursing homes, vitamin D, mental health, intervention study

## Abstract

**Objectives:**

Older people are recommended to take oral vitamin D supplements, but the main source of vitamin D is sunlight. Our aim was to explore whether active encouragement to spend time outdoors could increase the levels of serum 25-hydroxyvitamin D (25(OH)D) and increase the mental well-being of nursing home residents.

**Design:**

A cluster randomized intervention trial.

**Setting:**

Nursing homes in southern Sweden.

**Participants:**

In total 40 people >65 years.

**Intervention:**

The intervention group was encouraged to go outside for 20–30 minutes between 11 a.m. and 3 p.m. every day for two months during the summer of 2018.

**Measurements:**

We analyzed serum 25(OH)D before and after the summer. Data from SF-36 questionnaires measuring vitality and mental health were used for the analyses.

**Results:**

In the intervention group, the baseline median (interquartile range (IQR)) of serum 25(OH)D was 42.5 (23.0) nmol/l and in the control group it was 52.0 (36.0) nmol/l. In the intervention group, the 25(OH)D levels increased significantly during the summer (p=0.011). In the control group, there was no significant change. The intervention group reported better self-perceived mental health after the summer compared to before the summer (p=0.015). In the control group, there was no difference in mental health.

**Conclusion:**

Active encouragement to spend time outdoors during summertime improved the levels of serum 25(OH)D and self-perceived mental health significantly in older people in nursing homes and could complement or replace oral vitamin D supplementation in the summer.

## Introduction

Vitamin D deficiency is common in older people living in nursing homes in the Scandinavian countries (1, 2, 3, 4). The Swedish Food Agency recommends that all people over 75 years take an oral daily vitamin D supplement of 20 micrograms to ensure an adequate intake (5). A Dutch study with community-dwelling older adults showed that sun exposure, vitamin D intake and vitamin D-related genes substantially contributed to the variation in serum 25-hydroxyvitamin D (25(OH)D), and of those three factors, sun exposure was the most important (6). Another study of older nursing home residents showed that irradiation from an artificial UVB source was as effective as oral vitamin D supplementation for increasing the levels of serum 25(OH) D (7), but artificial UV radiation is not a standard care in nursing homes. Previous intervention studies about the effect of increased natural sunlight exposure on the 25(OH)D levels in older people in residential care have shown inconsistent results. Reid et al. studied the effect of 0, 15 and 30 minutes of daily sun exposure over four weeks in 15 older people in residential care in Auckland, New Zealand (8). They found increased vitamin D concentrations and positive effects on the calcium metabolism in the group with 30 minutes sun exposure, suggesting this as a method to prevent osteomalacia. Sambrook et al. studied the effect on vitamin D status and fall incidence of an intervention with sunlight exposure for 30–40 minutes, five days/week over 12 months and calcium supplementation in older people in residential aged care facilities in Sydney, Australia. The adherence to the intervention was low, and overall, showed no significant increase in serum 25(OH)D or reduction in fall risk (9). In a sub study, the UV doses were estimated. The UV dose had a small, but significant positive effect on the vitamin D levels after six months only if the sunlight sessions started in spring or autumn, but still the 25(OH)D levels were below 50 nmol/l after the summer (10).

To our knowledge, there have been no previous intervention studies confined to older nursing home residents at northern latitudes on the effect of increased natural sunlight exposure on vitamin D levels. Therefore, our primary aim was to explore whether an intervention with active encouragement to spend time outdoors during summertime could increase the levels of vitamin D in people living in Swedish nursing homes. Furthermore, we aimed to investigate whether the intervention could increase the mental well-being of the study population.

## Methods

This was a cluster randomized intervention trial confined to nursing home residents in Jönköping in southern Sweden. To be included in the study the residents had to live in the selected nursing homes, be expected to stay in the nursing homes over the summer and be > 65 years. Exclusion criteria for participants were: planned to have a short-term stay, undergoing palliative care/with short expected survival, other reasons to believe that the resident was not expected to go outdoors, and inability to give informed consent to the study. Residents with severe dementia were not asked to participate. We obtained written informed consent from all participants.

The study was approved by the Regional Ethical Review Board, Linköping, Sweden and complied with the principles in the Declaration of Helsinki.

### Measurements before the summer

The intervention group consisted of individuals living in two selected small nursing homes and the control group were individuals living in another selected large nursing home. The study participants were consecutively included between April and May, 2018. Age, gender and smoking habits were registered. The participants' height was measured to the nearest centimetre and the weight to the nearest kilogram. The waist circumference was measured between the lowest rib and the hip bone to the nearest centimetre. The blood pressure was measured in a sitting position after five minutes of rest. The functional level was estimated with the second subscale Physical activity in the modified Norton scale (11). Data about medical history and current medication were collected from the medical records.

### SF-36

The participants answered the nine questions regarding vitality and mental health in the short form health survey SF-36, Table 1, which is a standardized questionnaire measuring self-reported health (12). The answers were compiled according to a standardized scoring protocol (13). The SF-36 has been evaluated in Sweden and found to be reliable (14).

**Table 1:** Questions on vitality and mental health from the short form health survey SF-36

**How much of the time during the past four weeks**	
1.	did you feel full of pep?
2.	have you been a very nervous person?
3.	have you felt so down in the dumps that nothing could cheer you up?
4.	have you felt calm and peaceful?
5.	did you have a lot of energy?
6.	have you felt downhearted and blue?
7.	did you feel worn out?
8.	have you been a happy person?
9.	did you feel tired?


The health concept of vitality was measured using questions 1, 5, 7 and 9, and mental health was measured using questions 2, 3, 4, 6 and 8; The items were assessed from 1 to 6: 1= all of the time, 2= most of the time, 3= a good bit of the time, 4= some of the time, 5= a little of the time and 6= none of the time.

### Laboratory methods

A venous blood sample was taken in the non-fasting state between April and May 2018, before the start of the intervention and was used for routine laboratory analyses at Ryhov County Hospital, Jönköping, Sweden. Serum 25(OH) D was analyzed with a 1-step delayed chemiluminescent microparticle immunoassay technique with an Architect i2000SR immunoassay analyzer, Abbott Laboratories, Abbott Park, Illinois, USA.

### The intervention

The participants in the intervention group were encouraged, orally and in writing, to go outside for 20–30 minutes between 11 a.m. and 3 p.m. every day for two consecutive months, starting on any day between May 15 and July 1, 2018. The staff were asked to continuously remind the intervention group to go outside on a daily basis regardless of the weather. The intervention group received written advice from the Swedish Radiation Safety Authority about sun protection. The control group lived as usual. All participants were instructed to record the days with time spent outdoors in a diary.

### Measurements after the summer

In September/October 2018, a venous blood sample for follow-up on serum 25(OH)D was drawn and the participants answered the SF-36 questionnaire regarding vitality and mental health again.

### Food

The number of times fish/seafood was on the menu in the nursing homes during the intervention period was recorded. In one of the nursing homes for the intervention group, fish/seafood dishes were served 56 times, in the other nursing home for the intervention group 52 times, and in the nursing home for the control group 58 times.

### Weather

We collected data on sunlight time from the Swedish Meteorological and Hydrological Institute, measured in Växjö, Sweden, located approximately 106 km from the nursing homes. Sunlight time was defined as the number of hours with direct solar radiation above 120 W/m2 measured with a contrast sensor. The summer of 2018 was sunny in southern Sweden. The number of sunlight hours was 352 in May, 308 in June, 357 in July and 188 in August (15).

### Statistics

We used IBM SPSS Statistics 25 (International Business Machines Corporation, New York, USA) for the statistical analyses. Non-parametric tests were used as the variables were not normally distributed and the population was small. A Mann-Whitney U test and a related samples Wilcoxon signed-rank test were used to compare median levels of continuous variables between groups. Regarding the results of SF-36, t-tests were used to compare mean levels of continuous variables between groups. We used the Chi-square test to investigate associations between categorical data. Fischer's exact test was used when more than 20% of the cells had an expected frequency below 5. Statistical significance was defined as p<0.05.

## Results

**Figure 1:**
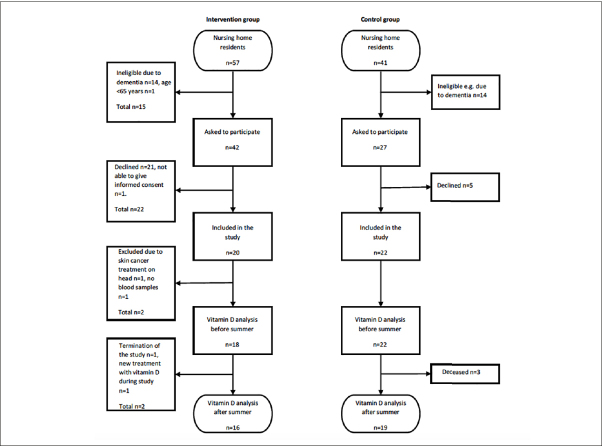
Flow chart of the study

We included 20 people in the intervention group and 22 people in the control group, as shown in Figure 1. Eighteen people in the intervention group and all of the 22 participants in the control group remained for analyses before the summer. The baseline characteristics of the study population are described in Table 2. There were no differences in age, sex, BMI, waist circumference, blood pressure, smoking habits or physical activity levels between the groups. The participants in the control group had lower plasma glucose than the intervention group. No differences were seen in levels of parathyroid hormone, haemoglobin, albumin-corrected calcium, creatinine or estimated glomerular filtration rate between the groups. There was extensive co-morbidity in the population but no differences between the groups regarding diagnoses or medication.

**Table 2:** Baseline characteristics of the study participants

	**Intervention group**	**Control group**	**p-value**
Number	18	22	
Age (years)	85.5 (12.0)	87.0 (10.0)	0.68
Male	44%	46%	0.95
BMI (kg/m2)	27.8 (8.9)	25.4 (6.2)	0.17
Waist circumference (cm)	109.5 (26.0)	95.0 (20.0)	0.28
Systolic blood pressure (mmHg)	120.5 (22.0)	125.0 (25.0)	0.84
Diastolic blood pressure (mmHg)	72.0 (10.0)	73.5 (18.0)	0.66
Smoking			0.26 ^a^
Current	17%	5%	
Previous	33%	18%	
Never	50%	77%	
Level of physical activity in the modified Norton scale			0.87 ^a^
Bedridden	0%	0%	
Chairbound	39%	32%	
Walks with help	6%	5%	
Ambulant	56%	64%	
Serum 25(OH)D (nmol/l)	42.5 (23.0)	52.0 (36.0)	0.20
PTH (pmol/1)	8.4 (7.7)	7.4 (4.8)	0.49
Haemoglobin (g/1)	122.0 (16.0)	122.0 (22.0)	0.58
Calcium, albumin-corrected (mmol/1)	2.37 (0.13)	2.36 (0.19)	0.60
Creatinine (/<mol/l)	82.0 (49.0)	86.0 (33.0)	0.24
eGFR(ml/min/1.73m^2^)	56.0 (26.0)	51.5 (23.0)	0.34
Plasma glucose (mmol/1)	6.2 (4.9)	5.8 (0.8)	0.026*
Frequency of people with diagnosis according to medical record:			
Ischaemic heart disease	28%	36%	0.56
Heart failure	56%	55%	0.95
Atrial fibrillation	33%	46%	0.44
Hypertension	44%	18%	0.07
Previous stroke/TIA	44%	23%	0.15
Diabetes	33%	14%	0.25 ^a^
Renal failure	33%	23%	0.50 ^a^
Known malignancy	6%	27%	0.11 ^a^
Osteoporosis	11%	27%	0.26 ^a^
Depressive/ anxiety disorder	61%	46%	0.32
Frequency of people with treatment with:			
Anti-hypertensive therapy	67%	59%	0.62
Anticoagulants	33%	46%	0.44
Platelet inhibition therapy	39%	41%	0.90
Statins	44%	32%	0.41
Oral anti-hyperglycaemic therapy	11%	0%	0.20 ^a^
Insulin	28%	9%	0.21 ^a^
Calcium substitution	11%	18%	0.67 ^a^
Vitamin D substitution	17%	18%	1.00 ^a^
Anti-depressant medication	61%	46%	0.32


Data are presented as medians and interquartile ranges or as percentages. The Mann-Whitney U test was used to compare median levels of continuous variables between groups. The Chi-square test was used to investigate associations between categorical data. The Fischer's exact test was used when more than 20% of the cells had an expected frequency below 5 (a). A p-value of <0.05 was considered significant (*). BMI, body mass index; 25(OH)D, 25-hydroxyvitamin D; PTH, parathyroid hormone; eGFR, estimated glomerular filtration rate; TIA. transient ischaemic attack.

### Serum 25(OH)D before the summer

For all participants, the median (interquartile range (IQR)) of serum 25(OH)D was 45.0 (28.0) nmol/l before summer. In the intervention group, the median (IQR) of serum 25(OH)D was 42.5 (23.0) nmol/l. In the control group, the median (IQR) of serum 25(OH)D was 52.0 (36.0) nmol/l. No difference in serum 25(OH)D was seen between the groups.

### Serum 25(OH)D after the summer

**Figure 2:**
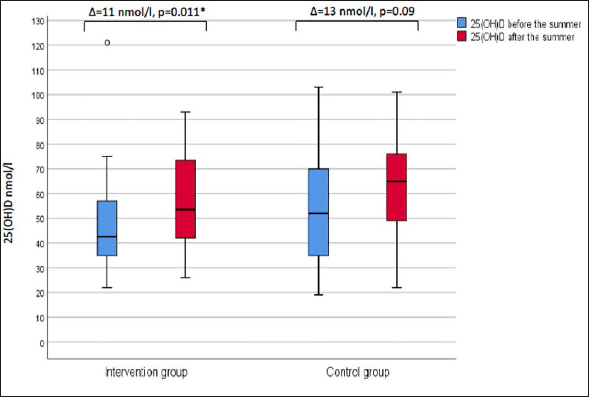
Changes in serum 25(OH)D during the summer for older people in nursing homes

For all participants, the median (IQR) of serum 25(OH)D was 64.0 (34.0) nmol/l after the summer. The serum 25(OH) D levels were significantly higher after the summer versus before the summer (p=0.004). The changes in serum 25(OH) D during summer are shown in Figure 2. In the intervention group, the median (IQR) of serum 25(OH)D was 53.5 (33.0) nmol/l and there was a significant increase compared to the levels before the summer (p=0.011). In the control group, the median (IQR) of serum 25(OH)D was 65.0 (35.0) nmol/l with no difference compared to before the summer. No difference in serum 25(OH)D was seen between the intervention group and the control group after the summer.

Box- and whisker plot (median, first and third quartile, range) of 25(OH)D. The related samples Wilcoxon signed-rank test was used to compare median levels of 25(OH)D before and after the summer. A p-value of <0.05 was considered significant (*). 25(OH)D, 25-hydroxyvitamin D

### SF-36

The results of the SF-36 are shown in Table 3. The participants in the intervention group reported better self-perceived mental health after the summer compared to before the summer (p=0.015). No difference was seen in vitality scores. In the control group, there were no differences in vitality or mental health scores.

**Table 3:** Results of SF-36

	**Intervention group**	**Control group**	**p-value**
*Vitality score*			
Before summer	51.9 (22.7)	46.6 (30.6)	p=0.54
After summer	58.8 (17.9)	50.8 (33.0)	p=0.40
p-value	p=0.15	p=0.17	
*Mental health score*			
Before summer	72.2 (20.3)	67.1 (27.3)	p=0.51
After summer	82.4 (16.4)	71.8 (29.1)	p=0.21
p-value	p=0.015*	p=0.09	


Data are presented as means and standard deviations. The independent samples t-test was used to compare mean levels in scores between the intervention group and the control group. The paired samples t-test was used to compare mean levels in scores before and after the summer. A p-value of <0.05 was considered significant (*).

## Discussion

In this intervention study confined to older people living in Swedish nursing homes, we found that active encouragement to spend time outdoors during summertime improved their serum 25(OH)D levels significantly. The serum 25(OH)D levels increased both in the intervention group and in the control group during the summer, but in the control group the difference was not significant. The intervention group reported improved self-perceived mental health after the summer compared to before the summer. However, there was no change in mental well-being in the control group. Compared to the Swedish population in the age group >65 years (mean score for vitality 66.4 and for mental health 80.3 (13)), our study participants had significantly lower vitality scores both before the summer (p<0.001) and after the summer (p=0.015). Regarding mental health, our study participants had significantly lower scores before the summer (p=0.007), but not after the summer (p>0.05) compared to the older Swedish population.

We know of two previous studies on the effect of natural sunlight exposure on vitamin D levels in older people in residential care, but both studies are from the southern hemisphere, one from Auckland, New Zealand, latitude 37°S (8) and the other from Sydney, Australia, 34°S (9). Our study was conducted in Jönköping, Sweden, latitude 57°N, where the climate is very different. Our results are in line with the results of the study of Reid et al. (8) but Reid's study was very small and of short duration, which is why the generalizability may be unclear. Our findings are in contrast to the Australian study, which showed no significant increase in serum 25(OH) D after a 12-month intervention period with increased sun exposure (9). However, a sub study showed that the UV dose had a small, but significant positive effect on the vitamin D levels after six months for those who started their sunlight sessions in spring or autumn (10). In Australia, dermal vitamin D production is possible all year while in Sweden it only occurs during the summer months, so a 12-month intervention period in Sweden would not be clinically relevant. The adherence to the intervention in the Australian study was poor and declined during the study. The duration of our study was two months, which might have helped to sustain the motivation to adhere to the intervention.

The strengths of this study are the randomized design and that older people living in nursing homes were included. This is an important, but difficult patient category to study, not least due to the high co-morbidity in this population, which was evident in our study. Many residents in the nursing homes were not eligible for participation due to severe dementia, and many residents declined participation, which significantly reduced the number of study participants. Despite this, we still found that the intervention with active encouragement to spend time outdoors was feasible and thus clinically relevant in this population. Ultraviolet radiation increases the risk of skin cancer, but the time in the sun needed for the desirable cutaneous synthesis of 25(OH)D is short. In this study, the time for the outdoor stay was set to 20–30 minutes per day and the participants received information about sun protection.

Although larger than several others in the field, a limitation of our study is its small size. A further limitation is the open label design, i.e. that the study participants and the staff were not blinded to the intervention.

What does this study add to the knowledge in the field? Firstly, to the best of our knowledge, this is the first study of frail older people showing that an intervention with encouragement to spend time outdoors resulted in both increased 25(OH)D levels and improved self-reported mental health. Secondly, the intervention is feasible, inexpensive and thus clinically relevant. However, from this study we cannot identify the reason for the improved mental health in the intervention group, nor tell if there is any association between 25(OH)D and mental health. Possible explanations for the improved mental health after the summer, apart from increased 25(OH)D level, may include increased social interaction and more physical activities when spending time outdoors.

Both the intervention group and the control group reached serum 25(OH)D levels above 50 nmol/l immediately after the summer, but it is doubtful if the achieved values would be high enough to maintain adequate levels throughout the consecutive dark season without oral vitamin D supplements. Though, during the summer months active encouragement to increased time outdoors, leading to increased natural sunlight exposure and increased vitamin D levels, could be a complement to or a replacement of oral vitamin D supplementation. This is appealing as polypharmacy is a common problem for many older people (16). Other possible effects of increased time outdoors such as improved mental well-being and more social interaction could follow. If encouragement to spend time outdoors becomes a practical measure to increase the vitamin D levels in older people, it is important to have balanced information about the benefits and risks of sunlight exposure.

In conclusion, active encouragement to spend time outdoors during the summer improved serum 25(OH)D and self-perceived mental health significantly in older people in nursing homes and could be a complement to or a replacement of oral vitamin D supplementation in the summer. These results should be taken into consideration when issuing updated recommendations about the activities and staff numbers in nursing homes for older people.
